# Supplementary material for: Modeling glioblastoma heterogeneity as a dynamic network of cell states
Source: Mol Syst Biol. 2021 Sep 16;17(9):e10105. doi: 10.15252/msb.202010105 (PMC8444284; doi:10.15252/msb.202010105)
Supplement: Supplementary file 6 — Source Data for Figure 5 [file MSB-17-e10105-s004.zip › Figure5A_sourcedata/GSEA_3017/hallmarks_stateF.GseaPreranked.1621934628066/HALLMARK_MYOGENESIS.html]

Details for gene set HALLMARK\_MYOGENESIS[GSEA]

|  || Dataset | state33017 |
| Phenotype | NoPhenotypeAvailable |
| Upregulated in class | na\_pos |
| GeneSet | HALLMARK\_MYOGENESIS |
| Enrichment Score (ES) | 0.48431548 |
| Normalized Enrichment Score (NES) | 2.4163704 |
| Nominal p-value | 0.0 |
| FDR q-value | 0.0 |
| FWER p-Value | 0.0 |
Table: GSEA Results Summary

  

Fig 1: Enrichment plot: HALLMARK\_MYOGENESIS      
 Profile of the Running ES Score & Positions of GeneSet Members on the Rank Ordered List

  

| PROBE | GENE SYMBOL | GENE\_TITLE | RANK IN GENE LIST | RANK METRIC SCORE | RUNNING ES | CORE ENRICHMENT || 1 | CDKN1A |  |  | 4 | 2.089 | 0.0865 | Yes |
| 2 | CLU |  |  | 5 | 1.852 | 0.1648 | Yes |
| 3 | SPARC |  |  | 52 | 0.929 | 0.1832 | Yes |
| 4 | NQO1 |  |  | 53 | 0.918 | 0.2220 | Yes |
| 5 | FST |  |  | 61 | 0.869 | 0.2556 | Yes |
| 6 | ITGB5 |  |  | 80 | 0.803 | 0.2814 | Yes |
| 7 | PDE4DIP |  |  | 82 | 0.794 | 0.3145 | Yes |
| 8 | CRYAB |  |  | 85 | 0.783 | 0.3467 | Yes |
| 9 | APP |  |  | 95 | 0.759 | 0.3747 | Yes |
| 10 | GNAO1 |  |  | 141 | 0.685 | 0.3833 | Yes |
| 11 | IGFBP7 |  |  | 164 | 0.643 | 0.4005 | Yes |
| 12 | DTNA |  |  | 176 | 0.617 | 0.4216 | Yes |
| 13 | GABARAPL2 |  |  | 232 | 0.568 | 0.4207 | Yes |
| 14 | SCHIP1 |  |  | 233 | 0.566 | 0.4446 | Yes |
| 15 | PRNP |  |  | 269 | 0.538 | 0.4515 | Yes |
| 16 | MRAS |  |  | 289 | 0.518 | 0.4648 | Yes |
| 17 | EIF4A2 |  |  | 380 | 0.478 | 0.4443 | Yes |
| 18 | PLXNB2 |  |  | 398 | 0.471 | 0.4565 | Yes |
| 19 | AK1 |  |  | 399 | 0.470 | 0.4763 | Yes |
| 20 | ATP6AP1 |  |  | 450 | 0.450 | 0.4727 | Yes |
| 21 | FHL1 |  |  | 467 | 0.445 | 0.4843 | Yes |
| 22 | PFKM |  |  | 547 | 0.416 | 0.4661 | No |
| 23 | CNN3 |  |  | 576 | 0.407 | 0.4706 | No |
| 24 | APOD |  |  | 636 | 0.389 | 0.4604 | No |
| 25 | MEF2C |  |  | 696 | 0.372 | 0.4494 | No |
| 26 | AGRN |  |  | 751 | 0.359 | 0.4401 | No |
| 27 | SPTAN1 |  |  | 815 | 0.344 | 0.4262 | No |
| 28 | COL6A2 |  |  | 855 | 0.335 | 0.4227 | No |
| 29 | RIT1 |  |  | 916 | 0.324 | 0.4092 | No |
| 30 | BHLHE40 |  |  | 928 | 0.321 | 0.4178 | No |
| 31 | MAPRE3 |  |  | 961 | 0.316 | 0.4167 | No |
| 32 | FGF2 |  |  | 964 | 0.316 | 0.4291 | No |
| 33 | AGL |  |  | 976 | 0.314 | 0.4374 | No |
| 34 | FKBP1B |  |  | 1000 | 0.310 | 0.4401 | No |
| 35 | SIRT2 |  |  | 1018 | 0.307 | 0.4454 | No |
| 36 | SORBS1 |  |  | 1068 | 0.297 | 0.4358 | No |
| 37 | LAMA2 |  |  | 1123 | 0.289 | 0.4235 | No |
| 38 | DMD |  |  | 1153 | 0.285 | 0.4224 | No |
| 39 | CKB |  |  | 1200 | 0.279 | 0.4134 | No |
| 40 | OCEL1 |  |  | 1271 | 0.272 | 0.3932 | No |
| 41 | ADCY9 |  |  | 1306 | 0.268 | 0.3891 | No |
| 42 | ITGB1 |  |  | 1521 | -0.265 | 0.3035 | No |
| 43 | HBEGF |  |  | 1583 | -0.282 | 0.2878 | No |
| 44 | TPM3 |  |  | 1780 | -0.353 | 0.2140 | No |
Table: GSEA details [plain text format]

  

Fig 2: HALLMARK\_MYOGENESIS: Random ES distribution      
 Gene set null distribution of ES for **HALLMARK\_MYOGENESIS**
